# Editorial: Insights in healthcare professions education: 2022

**DOI:** 10.3389/fmed.2024.1539195

**Published:** 2025-01-10

**Authors:** Lynn V. Monrouxe, Jacqueline G. Bloomfield

**Affiliations:** The University of Sydney School of Health Sciences, Camperdown, NSW, Australia

**Keywords:** interprofession education, underperformance, mentorship, feedback, competencies, simulation-based education

## Introduction

Welcome to the 2022 edition of “*Insights in Healthcare Professions Education”*—our curated Research Topic of 10 articles reveal the latest trends shaping the future of medical and healthcare education. In these articles from around the globe, we explore three interrelated themes: How healthcare education needs collaboration, the growing influence of technology in both healthcare and education, and learning and enhancing competency and managing workloads. Collectively, these articles highlight the delicate balance between leveraging cutting-edge digital tools and maintaining the human touch in training and practice. They emphasize the importance of hands-on learning, empathy, and person-centered care, alongside technology's increasingly important role in enhancing outcomes. With a focus on collaboration, mentorship, and proactive strategies, this collection offers fresh insights into building resilient health professionals and empowering patients—paving the way for a more sustainable healthcare future.

## Collaboration in healthcare education

Collaborative and interdisciplinary learning is an important evolution in healthcare education as Soemantri et al. point out. Indeed, having an effective measure of interprofessional competencies enables educators to understand what further interventions might be required for the nurturing and maintenance of collaborative practice in any given setting. As such they report on their work demonstrating the cross-cultural validity of the Chiba Interprofessional Competency Scale (CICS29) for measuring the interprofessional competencies of health professionals. Further, in this collaborative space, Ajjawi et al. explore the critical role of feedback in supporting underperforming trainees. Using a narrative review synthesis, these authors examined how learning and safety can be combined in the context of underperformance. They suggest that collaborative, trust-based feedback processes can foster emotional resilience and performance improvement for healthcare trainees.

Focusing on educating tomorrow's healthcare professionals to better understand the range of addictions and how best to treat them, Lundin and Hill call for collaboration between medical specialties, training organizations, and universities. They argue the essential nature of this collaboration would be to create a much-needed unified training framework that incorporates core competencies while adapting to evolving treatments, technologies, and legislation. Building on existing suggestions, more detailed work is now needed to define specific knowledge areas, with innovations like machine learning offering valuable tools to identify substance use issues and shape targeted learning goals.

Finally, a different type of collaboration, that of mentorship and career development, is highlighted by Hertling et al. They surveyed 43 medical schools and 1,516 students across Germany with the aim of understanding the scope of this practice. They found that just over half of medical schools in their sample offered mentoring programmes, with this being less common across surgical programmes (30%). Students reported that structured mentoring programmes were highly desirable and indicated a willingness to participate in them (including those with a surgical focus), agreeing they would have an impact on planning their future career trajectories.

Across the articles in this theme, we can see how the evolution of healthcare education is characterized by a push toward hybrid models, merging digital efficiency with experiential learning. Collaborative environments, mentorship, and effective feedback mechanisms are also crucial in nurturing skilled and emotionally resilient healthcare professionals.

## Technology in healthcare and education

Technological advancements are transforming healthcare education. Indeed, education in healthcare is continually adapting to modern demands: balancing digital innovations with traditional approaches. With this in mind, Vogt et al. examined seminars and practical learning of emergency medicine delivered via exclusively digital vs. traditional teaching formats. They found that hands-on training remains indispensable for acquiring clinical competencies in this context with, somewhat surprisingly, purely digital education leading to worse performance in students' final exams in comparison with competency-based onsite learning. Recker et al. reinforces this, highlighting the importance of integrating theory with structured, supervised, hands-on exercises when addressing the skill gaps in clinical practice around point-of-care ultrasound learning.

Role models are influential, this is not news. But what is new is the concept of role model-based digital interventions. Li et al. examined this very issue using a randomized experiment withmedical students (*n* = 36, 4,832) from 93 institutions across 30 provinces of China. The survey administered to the intervention group contained text and images of Zhong Nanshan, a role model who praised healthcare workers' pro-social behaviors in the fight against the COVID-19 pandemic via mainstream social media. The control group completed the same survey without this information. Using a pre-post career commitment measure, the researchers found that career commitment enhanced significantly in the intervention group as a whole, with the male and senior students being influenced to a greater extent than both their female and junior colleagues.

Undoubtably, technology is a powerful enabler, providing solutions for scalable education, real-time feedback, and effective role modeling. However, as Vogt et al. and Recker et al. highlight, technology must support—not replace—human elements of empathy, collaboration, and direct interaction.

## Enhancing competency and managing workloads

With the aim of developing competent surgeons, Suzuki et al. explored the mental workload they experienced when performing endoscopic sinus surgery (ESS) under controlled simulation settings using 3D-printed sinus models. Their findings demonstrated that aspects of mental workload, such as temporal demand, effort, and perceived performance, are closely tied to surgeons' skill levels and can improve through repetitive training. However, other factors, like physical and mental demand, may worsen with experience, highlighting the nuanced nature of skill acquisition in high-stress environments. This research emphasizes the importance of routine monitoring of surgeons' mental health and stress levels during training, advocating for strategies to mitigate burnout and support mental resilience.

Competency is also the focus of Rakab et al.'s study. This article shifts focus to the diagnostic competencies of resident doctors, specifically in electrocardiographic (ECG) interpretation. Conducted across eight Arabic countries, the study reveals a correlation between years of residency and ECG interpretation proficiency, alongside a lack of training in this area. This work complements the skill-level discussions in Suzuki et al., demonstrating how competency evolves over time and through targeted educational interventions. Together, they highlight the need for structured training programs, simulation-based learning, and progressive skill-building frameworks to ensure healthcare professionals are well-prepared for the complexities of their roles.

The balance that clinical pharmacy faculty members strike between academic duties and direct patient care in Saudi Arabia was examined by Korayem et al.. In this study, faculty members reported dedicating the highest percentage of their time to clinical education, with patient care receiving less attention. This was due to barriers such as unclear practice policies and administrative constraints. As with the previous two articles in the theme, there is an emphasis on the importance of optimizing time and resources to foster professional growth while maintaining high standards of patient care and education. This work also complements the discussions in this theme by exploring how healthcare professionals and educators allocate their time and effort. While Suzuki et al. and Rakab et al. emphasize the importance of skill acquisition, Korayem et al., probe into the practical challenges of balancing educational duties and direct patient care. The findings collectively suggest the need for workload models and policies that prioritize both professional development and patient outcomes.

## Summary

Having summarized this Research Topic across three essential themes, a cross-cutting narrative develops: that of the importance of integrating human-centered approaches with innovative tools to enhance both learning and practice. The evolution of healthcare education thrives on collaborative frameworks, as demonstrated by the critical role of interprofessional competencies, trust-based feedback, and mentorship. Such initiatives foster emotionally resilient and well-prepared professionals, while interdisciplinary training unites diverse specialties to address complex challenges such as addiction management. Simultaneously, technological advancements provide scalable solutions and transformative possibilities, exemplified by digital role-modeling and point-of-care ultrasound training. However, as we have seen, technology must complement, not replace, experiential learning and the empathy-driven interactions essential in healthcare. Finally, competency development and workload management are pivotal to sustaining excellence. Simulation-based training, structured educational programs, and balanced workload models ensure that healthcare professionals can meet evolving demands without compromising their mental wellbeing. Together, these themes highlight an holistic approach to healthcare education—one that merges collaboration, technology, and strategic competency-building to prepare adaptable and resilient professionals for the future of healthcare.

